# Editorial: Novel Combination Therapies for the Treatment of Solid Cancers

**DOI:** 10.3389/fonc.2021.708943

**Published:** 2021-06-18

**Authors:** Nehad M. Ayoub

**Affiliations:** Department of Clinical Pharmacy, Faculty of Pharmacy, Jordan University of Science and Technology (JUST), Irbid, Jordan

**Keywords:** combination, resistance, repositioning, synergy, efflux transporters

## 

The concept of combination therapy was first introduced in 1965 when Emil Frei et al. launched the first-ever combination chemotherapy in pediatric patients with acute leukemia (1). The success of this combination therapeutic approach had remarkably changed the landscape of clinical oncology ever since (2). Consequently, much emphasis in cancer research was directed to investigating combination therapies that target different pathways to generate a favorable anticancer activity (3). In line with this, advancements in cancer cell genomics, epigenomics, transcriptomics, and proteomics have paved the way to identifying new molecular targets and the development of selective targeted anticancer therapies (4). Targeted therapies have substantially expanded the options for combinational anticancer treatments that can be combined with other targeted therapies or chemotherapeutic drugs (5).

The combination of anticancer therapies is clinically appealing for several reasons. Firstly, combination therapy improves treatment outcomes and results in superior therapeutic effects, especially when a synergistic anticancer activity is achieved (6). Secondly, the combinational approach overcomes clonal heterogeneity which is further associated with improved response rates (7). Thirdly, combined drug regimens reduce the toxicity of the regimen as it allows using individual drugs at reduced dosages at maintained therapeutic efficacy (6). Another advantage of combination therapies is reducing the emergence of drug resistance (6). In this context, combination therapy enables concurrent targeting of several molecular pathways essential for cancer cell survival and abolish cellular mechanisms associated with adaptive resistance (8). Despite the advantages of combination cancer treatments, several challenges accompany the development and utilization of combined therapies. A challenging aspect of combination therapies is the potential drug interactions and the pharmacokinetics of co-administered agents that could influence the therapeutic activity of the regimen (2, 9). Besides, the administration of suboptimal doses of drugs in the combination may be necessary to avoid toxicity (9). The definition of synergism is inconclusive, particularly in clinical studies, and its prediction is challenging (10).

Historically, the development of most drug combinations was conducted using empirical experimental or clinical settings (7, 8). In such a case, a detailed mechanistic analysis is rarely performed for the prediction of effective combinations (7). Therefore, the development of strategies for the prediction and identification of combinations that exhibit synergy is imperative (7, 8). In this regard, Narayan et al. used a new approach called ‘*drug atlas*’ to identify novel synergistic combination therapies (8). This strategy allows the identification of independent processes for which the tumor might be particularly vulnerable when attacked by two drugs on a pan-cancer scale. A restrictive combination of drugs is another approach that is gaining attention in cancer therapy (3). This restrictive approach is based on the differences between cancer cells and normal cells and focuses on strategic dosing and drug administration to spare normal cells while targeting cancer cells (3, 11). Besides, Tolcher et al. have demonstrated the use of the ‘*CombiPlex*’ technology platform as a valuable tool for developing drug combinations to predict the likelihood of clinical activity of anticancer therapies (9). The *CombiPlex* platform improves drug combinations by identifying synergistic drug ratios and directing drug exposure to target tissues (9).

In this Research Topic, several studies have investigated the impact of novel anticancer combinations and strategies in the treatment of solid cancers. Colombo et al. studied the anticancer activity of birinapant, an inhibitor of the inhibitor of apoptosis proteins, in non-small-cell lung cancer. The activity of birinapant was demonstrated in liver kinase B1 (LKB1)-deleted clone but not LKB1-wild type cancer cells. In addition, the combination of birinapant with the p38 inhibitor, ralimetinib, restored the sensitivity of LKB1- and KRAS-mutated cell lines to birinapant. In another study, Mortensen et al. investigated the impact of the novel heat shock protein 90 (HSP90) inhibitor, onalespib to enhance the activity and reverse resistance of cisplatin in ovarian and head and neck cancer cells. The results of the study showed that the combination of onalespib and cisplatin restored therapeutic activity and enhanced the antiproliferative, antimigratory, and apoptotic effects of the chemotherapeutic drug. Shi et al. showed that sphingosine kinase 2 (SphK2) mediated regorafenib resistance in hepatocellular carcinoma through NF-kB and STAT3 activation. The authors also reported that sensitivity to regorafenib was restored with the combination of regorafenib and the SphK2 inhibitor ABC294640 in both *in vitro* and xenograft animal models of hepatocellular carcinoma. Xu et al. showed that the combination of the HSP90, SNX-2112 with the knockdown of STAT3 is associated with enhanced antiproliferative and apoptotic anticancer activity in esophageal cancer stem-like cells in culture and animal models.

The multidrug resistance of cancer cells is strongly linked to the overexpression of ATP-binding cassette (ABC) efflux transporters (12). In this Research Topic, Yang et al. and Wu et al. evaluated the use of ABC efflux transporter inhibitors as chemosensitizing agents to improve the activity of anticancer drugs. Yang et al. demonstrated the inhibitory effect of sitravatinib, a broad-spectrum tyrosine kinase inhibitor, on ABCG2 efflux transporters. Sitravatinib treatment blocked the efflux function of ABCG2 efflux transporters and restored the antineoplastic effect of various anticancer drugs known as ABCG2 substrates. In a second study by Wu et al., nedisertib (M3814), a potent and selective inhibitor of DNA-dependent protein kinase, attenuated the efflux activity of ABCG2 transporter without affecting the expression or cell surface localization of the pump. Nedisertib treatment increased the accumulation of the ABCG2 substrate drugs mitoxantrone and doxorubicin restoring their sensitivity in cancer cells.

Drug repurposing (also known as drug repositioning) is an increasingly recognized therapeutic approach in cancer therapy. Drug repurposing utilizes existing non-cancerous drugs to be used for cancer treatment (3). This approach is very attractive as it utilizes FDA-approved pharmaceutical agents with known safety and pharmacokinetic profiles as a source of new anticancer drugs at a reduced financial burden (3, 13). Li et al. found that aspirin inhibited proliferation and promoted apoptosis of lung and breast cancer cells. In addition, the authors reported that aspirin treatment delayed and overcame resistance to targeted therapy using *in vitro* and *in vivo* models. Hsu et al. demonstrated a synergistic anticancer activity for the combination of sildenafil, a phosphodiesterase inhibitor, and vincristine treatment against castration-resistant prostate cancer (CRPC). The authors showed that sildenafil synergistically potentiated vincristine-induced mitotic arrest and mitochondrial damage *in vitro* and synergized with vincristine on suppressing tumor growth in a xenograft animal model of CRPC. In a systematic review by Zhang et al., the cardioprotective effect of enalapril against anthracycline-induced cardiotoxicity was examined across 626 studies. Preliminary evidence showed that enalapril treatment was associated with reduced cardiac enzymes and improved left ventricular ejection fraction in cancer patients treated with anthracyclines.

In a meta-analysis by Li et al., the authors evaluated 12 randomized controlled trials for the clinical effectiveness of combining cetuximab treatment with chemotherapy for treating metastatic colorectal cancer (mCRC). They revealed an improved progression-free and overall survival for the combination of cetuximab with chemotherapy in wild-type KRAS patients. In a review by Peng et al., the combination of immunotherapy, particularly the immune checkpoint inhibitors, with other drug therapies or radiation was discussed as a novel approach in the treatment of bladder cancer. In this e-book, a phase II study by Urbani et al. evaluated the impact of dacarbazine treatment with peptide-based vaccination in combination with IFN-α2b in melanoma patients. No significant differences were observed between patients who received or did not receive dacarbazine treatment for relapse-free and overall survival. Căinap et al. conducted a retrospective analysis for patients with mCRC who were treated with bevacizumab as first- or second-line therapy and who received bevacizumab beyond the first progression (BYP). They report that doubling the dose of bevacizumab BYP improved overall survival in mCRC patients, and that bevacizumab was a suitable partner in combination with both oxaliplatin- and irinotecan-based regimens.

The use of combined chemotherapy becomes the standard practice in medical oncology. Taking into consideration the tremendous number of available chemotherapeutic and targeted anticancer drugs, the prediction, and development of novel drug combinations is a challenging task. Hence, it is mandatory to explore the tools necessary to predict the combinations with synergistic anticancer activity. Articles in this Research Topic contributed to the field of novel drug combinations in several aspects including the combinations intended to overcome cancer resistance and enhance anticancer drug activity, repurposing of drugs in combination regimens, and providing insights from human studies on novel combinational approach. Collectively, the future of novel combinations to treat solid cancers is promising with endless potentials for combination therapies on the horizon.

## Author Contributions

The author confirms being the sole contributor of this work and has approved it for publication.

## Conflict of Interest

The author declares that the research was conducted in the absence of any commercial or financial relationships that could be construed as a potential conflict of interest.
